# Frankenstein 2.0.: Identifying and characterising synthetic biology engineers in science fiction films

**DOI:** 10.1186/2195-7819-9-9

**Published:** 2013-10-01

**Authors:** Angela Meyer, Amelie Cserer, Markus Schmidt

**Affiliations:** Organisation for International Dialogue and Conflict Management (IDC), Vienna, Austria; Biofaction KG, Vienna, Austria

**Keywords:** Synthetic Biology, Films, Movies, Science fiction, Scientists, Bio-engineers, Stereotypes

## Abstract

Synthetic biology (SB) has emerged as one of the newest and promising areas of bio-technology. Issues typically associated to SB, notably in the media, like the idea of artificial life creation and “real” engineering of life also appear in many popular films. Drawing upon the analysis of 48 films, the article discusses how scientists applying technologies that can be related to SB are represented in these movies. It hereby discusses that traditional clichés of scientists in general tend to be sublated by new stereotypical characterizations. These reflect real trends in bio-technological research such as SB, especially the increased relationship between science and industry. Frankenstein 2.0. looks less like the old, genius yet mad scientist, and follows a more entrepreneurial than academic spirit.

**Electronic supplementary material:**

The online version of this article (doi:10.1186/2195-7819-9-9) contains supplementary material, which is available to authorized users.

## Introduction

On 20 May 2010, researchers at the J. Craig Venter Institute announced the creation of the first synthetic bacterium, whose genome had entirely been synthesised in the lab. At a press conference, biologist Craig Venter stated: “*This is the first self-replicating species that we’ve had on the planet whose parent is a computer*.” (ABC news, 2010). Only a few weeks later, the movie Splice was released in the United States (US). The film tells the story of two young scientists who engineer new synthetic creatures in the lab by combining DNA from different organisms. The concomitance of Venter’s alleged breakthrough in synthetic biology (SB) and the movie’s start in the US cinemas at almost the same time can certainly be seen as pure coincidence. Although it goes without doubt that the film makers have largely been inspired by research currently done in the field of SB and even advised by a group of scientists. Nevertheless, such parallels between fiction and reality as exemplified here are very likely to influence the awareness and perception by the audience of this new and emerging field of biology (see e.g. Gerbner 1987; Frayling 2005; Keppler 2006; Diken and Laustsen 2008; Kirby 2011). In particular, it can be assumed that especially the safety and ethical questions raised by Splice as well as the final catastrophe carry significance for spectators who had followed the breaking news in the media a couple of weeks before.

Drawing on results from the Austrian research project Cinema and Synthetic Biology (CISYNBIO),^a^ this article sets out to discuss and analyse how SB is taken up by popular movies. Focussing in particular on SB scientists and engineers as film characters, the central question addressed is: How are these scientists represented and depicted in science fiction films? To discuss this question and understand its implications for the public perception of this emerging field of research, the article gives specific emphasis to the use of stereotypes and asks how these have changed. Which new stereotypes can be found in science fiction films featuring SB scientists as characters? What aspects and phenomena do these images refer to and what do they hence express? Do these stereotypes sublate traditionally used ones or do they still partly resemble the traditional fictional image of the scientist? And if some aspects of traditional scientist stereotypes can still be found in some films, do these, consequently, get a new meaning? And how can this sticking to old patterns be explained?

The main hypothesis discussed is that images and characteristics used to depict SB scientists in modern science fiction films particularly emphasise a shift from a purely academic to an increasingly industry-oriented and entrepreneurial spirit. By doing so, these films convey an image that is less one of an individually acting genius but slightly mad or bad scientist, in the tradition of Dr. Frankenstein or Dr. Moreau, but more one of a modern, contemporary scientist working in an industry related scientific environment, close to how the media tend to portray SB engineers.

### SB – Increasing public awareness on a promising field of biological research

#### From a century old idea to an emerging bio-technology

SB is an emerging area of bio-technology that consists in the “*design and construction of new biological systems not found in nature. It aims at creating novel organisms for practical purposes but also at gaining insights into living systems by re-constructing them*” (Schmidt et al. 2009b). SB therefore sits at the interface between biology and engineering, representing a true interdisciplinary field with different scientific cultures. The basic idea of SB itself is already 100 years old. The term “*biologie synthétique*” was first mentioned by French biologist and physician Stéphane Leduc (1912). After his research was strongly criticised and refused by the Paris Academy of Science (Tirard 2009), the idea disappeared – only to be brought back on the table by scientists in the 1970s and again most recently at the beginning of the 21st-century (Danielli 1972; Arkin and Endy 1999; Knight 2005; Campos 2009). By applying engineering principles to biology, SB goes one step further than “conventional” bio-technology and opens new opportunities, notably by producing engineered cells and biological systems with novel or optimised functions.

#### A topic for the media and playground for science fiction

Besides an increase in number of SB researchers, students and scientific publications in recent years (Kronberger et al. 2012; Oldham et al. 2012), SB has started to be apprehended by a wider non-scientific audience too (see for the US: Rejeski 2010; and for Europe: European Commission 2010). Since the 1990’s, a number of pioneering research outcomes such as the cloning of a sheep in 1996 and the completion of the Human Genome Project in 1999 have been generating an increase in public interest in bio-technologies in general (Dinello 2005). Although rather sporadically, the debate about SB potentials and risks is today also reflected by the public media, mainly when major research breakthroughs are made (Rejeski 2010). Newspapers and magazines reporting on SB often do so by pinpointing its utopian potentials and qualities (Cserer and Seiringer 2009; Hellsten and Nerlich, 2011; Lehmkuhl 2011). In many cases, journalists associate SB with the idea of creating artificial life or generating new creatures following a Lego bricks approach. Such a link of course deviates from the real current SB research mainly focused on possible applications in fields such as the production of biofuels, biomaterials, as well as bioremediation (Schmidt 2012).

SB and SB related technologies have also found their way into science fiction. Here as well, the link is made in many cases between SB and the ability to artificially create life. Whereas in films, and in fiction in general, a particularly common associations has long been that between artificial generation or manipulation of living organisms and metaphors of sorcery, myths, centuries-old stories or legends, especially recent films tend to refer to modern bio-technologies to underpin their ideas of artificial life. To provide a setting for the creation of supernatural characters, many film makers toy around with either the newest or imaginable findings in bio-technology, conceive sophisticated labs and characterise bio-engineers at work, or make their protagonists explain complex bio-technological concepts and applications. While applying considerable poetic freedom, these directors propose a – fictional – impression of SB related research to their audience. Especially, they emphasise the idea that this research may one day result in novel life forms. Some films also provide a fictive preview of the future, such as Blade Runner, Code 46 or The Island, imagining effects and consequences bio-technological breakthroughs could one day have on social and socio-political developments.

This, as a consequence, leaves us with the question what perception and understanding of SB does the audience finally take away from these films.

#### Understanding the role of Hollywood for SB public perception

Several authors have analysed the representation in fiction of science or scientists in general (Gerbner 1987; Haynes 1993,2003; Weingart et al. 2003;2005; Frayling 2005). Few studies exist on the way science fiction takes up concepts, issues and approaches associated with bio-technologies and more precisely with SB and the application of engineering principles to biology.

For several reasons, research in this specific field is however important. As underlined by Christopher Frayling, we have to admit that “the gap between specialised knowledge and public understanding lies at the root of most fictional representations of the scientist (…), a gap (that) has usually been filled by stereotypical representations of one kind or another.” (Frayling 2005: 11). In other words, fiction uses this gap by proposing fictive impressions and images of science to a public that is otherwise far away from the scientific world. Given the novel character of SB research, lay knowledge remains particularly limited and sources of information are scarce. If SB has found its way into the public media, it still only sporadically appears in magazines and newspapers. In addition, these articles rather emphasise the sensational aspects about new findings than provide scientific facts and information. Also, SB is rarely, if ever, taught at school. If, in return, SB relevant aspects and ideas are treated in popular science fiction films, these become a major source of information and are hence likely to influence the public awareness, understanding and perception. Although the impact of showing SB and SB related applications in films on public perception is still largely unexplored, such an assumption is fostered by the fact that, besides the internet, films and television indeed present the primary information source for scientific topics by most Europeans and US Americans (EUROBAROMETER 2007; National Science Foundation (NSF) 2012).

In view of this role films are playing in terms of communicating science and raising awareness, we therefore need to understand what – positive or negative – images are conveyed, what expectations or fears are raised, as these are likely not only to contribute to the public perception of this new field of research and its findings but also to underpin any relationship of trust or distrust between science and society. Furthermore, by linking SB to the capacity to artificially create life, films touch upon widely debated ethical questions and concerns: is it morally permissible to create life in a test-tube, to interfere into natural processes or to create human bodies for spare parts? By drawing scenarios and imagining – either positive or negative – effects and consequences of bio-technological research, these films take up this debate and may hereby be of influence for their audience’s ethical assessment of new and emerging sciences (Dinello 2005).

Asking about how people perceive and understand science, based in our case on representations in movies, makes us emphasise the sociological dimension of research and hence understand research and science as a social activity (Mouton 1996; Amann 1991 Richter 1995). Research is not accidental but carried out in view of accomplishing a specific result, which, in turn, has some effects and impact on the society. Emphasising this sociological dimension brings us to focus on the actors behind research: in our case the SB scientist or engineer.

### Analysing SB aspects in movies

In total, we have analysed 48 movies that relate in any way to the idea of artificial life creation (see Additional file 1: Table S1). For their selection, we have conducted a key word research in online-film databases and compared available plot descriptions with a list of pre-defined issues, including “*artificial/ synthetic life*”, “*life creation*”, “*bio-engineering*”, “*bio-technologies*”, “*DNA*”, “*synthesis*”, and “*genome*”. In order to ensure global relevance of the analysed movies, we have included films only, if – according to box office databases, such as http://www.worldwideboxoffice.com – they have made more than 10 million US$ worldwide (see Additional file 1: Table S1). We have used this selection criterion as a proxy for the impact and popularity among the general public. Exceptions have only been made for classics, such as Fritz Lang’s Metropolis or Paul Wegener’s Golem, wie er in die Welt kam.

Among the selected films, we have identified 35 that directly refer either to key concepts of SB or to aspects close to the idea of applying engineering principles to biology in one way or the other.

To guide our selection of relevant films, we have used a pre-defined list of key words and concepts related to SB (See Table 1).

**Table 1:** **Overview of SB subfields and related keywords**

Subfield	Keywords
Constructing (natural) DNA	Synthetic DNA, constructing viruses in the lab, resurrecting extinct animals (e.g. mammoth) by reconstructing their DNA
Reconstructing organisms using natural bio-parts	Re-programming life, tuning life, biological parts, biobricks, engineering life, designing and constructing life
Minimizing natural organisms (top-down)	Reduced life, minimal life, simple life, minimal genome
Constructing whole cells or parts thereof	Creating life from scratch, artificial life, synthetic life, protocells, vesicles
Creating alternative/unnatural lifeforms	Alien life forms, unnatural life forms, weird life, shadow life, completely new life, unknown life, artificial life, second life

Based on O’Malley et al. (2008), Schmidt et al. (2009a), Deplazes (2009), Schmidt and Pei (2011).

For the selection of films, the focus has deliberately been chosen rather broad for several reasons. First and foremost, a concrete reference to “*synthetic biology*” as such could not be found in any film. Even in films that more or less clearly refer to SB techniques, the term itself is never used. Several films even put these technologies in relation with other, more “*conventional*” bio-technology or genomics applications, such as cloning or gene mutations, although there is an evident link to SB techniques. The selected 35 films clearly either mention concepts (e.g. “*engineering genes*”, “*DNA synthesis*” or “*synthetic DNA*”) or show products (e.g. synthetic or hybrid creatures from the lab, synthetic viruses, or characters with synthesised DNA and new biological functions) that are associated with SB, both in science and in the media. For comparative purpose, we decided to further include the remaining 13 movies that address the idea of artificial life creation, by considering different approaches and technologies, such as robotics, cybernetics or conventional cloning.

It is interesting to note that these films are mainly dealing with the application of SB or related bio-technologies to humans or animals. Applications much closer to real SB research, such as for instance to plants are more or less neglected, with the exception of Dr. Hineman’s intelligent plants in one of our comparative films, Minority Report. We could also find some cases of artificial virus creation, with the Resident Evil sequels as particularly significant example.

In each of the selected films, relevant sequences have been identified, transcribed and paraphrased, making a total of 470 sequences lasting between a few seconds and some minutes each. For the analysis, these sequences have been classified into four categories. On the one hand, the definition of these categories acknowledges our understanding of science as a social activity, as discussed above. On the other hand, our intention has been to provide an analysis of the representation of SB science in science fiction that is as broad as possible and covers as many different aspects of scientific research as possible. With these two considerations in mind, we have decided to conceptualise research as a process, that starts with the development of a research idea and a theory, is based on certain expectations and motivations, and leads to the production and application of results. Accordingly, we have classified the selected sequences, depending on whether they address (1) scientific ideas and theories, (2) intentions and motivations, (3) products and productions, and (4) the application of the products and their impact on their environment. In order to establish a link between the analysed films and the reality, for each of these four categories, we have identified subcategories reflecting the current debate about SB and reassigned the sequences accordingly. This, on the one side, has allowed us to compare the recurrence of SB relevant elements in the selected films. On the other side, it has helped us to assess the relevance of the films for raising public awareness and for the perception of SB. To define these subcategories, we have referred to public press analyses on commonly used and discussed pictures of SB, such as the one carried out by Cserer and Seiringer (2009).

### Results: Five categories of SB scientists

Analysing the way SB scientists and engineers are represented in the selected science fiction films, we have tried to cluster the characters according to their professional background and profile taking as main criteria the degree of independence in their work. This approach has been motivated by our intention to analysis the prevalence of the image of the individually acting genius but slightly mad or bad scientist, in the tradition of Dr. Frankenstein or Dr. Moreau, as outlined in our hypothesis, versus the emergence of a modern, contemporary scientist working in an industry related scientific environment. A first observation has been that we can divide the characters into two groups: those still acting on their own – and hence appearing more in the tradition of the classical single professor image – and those working under an assignment or even within a whole team. This observation has led us to classify the portrayed characters into 5 categories, including three types of individually acting researchers that however differ according to their freedom to act, not further illustrated research teams and faceless companies or regimes (See Table 2).

**Table 2:** **The analytical categories used to discern different scientific actors in movies**

Description of scientific actor			Category
Individual researchers	Independent bioengineers		1
Individual researchers	Employed bioengineers	Rebellious employees (do not act in accordance with their assignment)	2
Individual researchers	Employed bioengineers	Loyal employees (act in accordance with their assignment)	3
Research teams			4
Companies and regimes			5

#### Individual researchers

*Independent bioengineers.* In the 35 analysed movies that refer to aspects or elements associated with SB, we have found eight portrayed scientists working more or less independently. Our main selection criterion has been that they do research either on their own behalf or upon an order they have got, sometimes receiving some external funding, but without being employed by any institution. They hence appear as somehow “*freelance bioengineers*”, pursuing their personal projects. This criterion has been applicable, although in quite different settings and contexts, to the following characters: Dr. Moreau in the 1996 adaptation of H.G. Wells’ novel The Island of Doctor Moreau; William Stryker in X-Men Origins: Wolverine; Dr. Wells in Godsend; Seth Brundle in the 1986 adaptation of The Fly; Dr. Darquandier in Babylon A.D.; a not further specified scientist in Blade II; Dr. David Banner in Hulk (after being dismissed from the US army); and Robert Neville in I am Legend. In the 13 comparative films, we have been able to identify seven individual characters who work on artificially creating, designing or modifying living organisms, by applying approaches and technologies other than SB: Dr. Frankenstein in the adaptation from 1994, Rabbi Löw in Golem, C. A. Rotwang in Metropolis, Claire Wellington in The Stepford Wives, Dr. Hineman in Minority Report, Rupert Burns in Bicentennial Man and Magneto in X-Men.

*The rebellious employee*. As a second group, we have identified individual scientists who work as (permanent) employees but, often secretly, pursue their own projects. A major criterion for this type of scientist has been that these characters are presented as own personalities, often in the position of team leader. Unlike independent bioengineers, their status as employed scientists generally places them in a situation where they have to execute their employers’ orders. Rebellious employees are aware of their assigned duties. However, they perceive these duties as contradictory with their personal ambitions. So they decide to pursue their own ideas, without informing or even against their employers’ interests. In the 35 films with SB reference, we could identify eight examples for this situation: Elsa and Clive in Splice, Susan McAlester in Deep Blue Sea, Dr. David Banner in Hulk (before being dismissed), Samuel Sterns in The Incredible Hulk; Grace Augustine in Avatar, as well as Dr. Isaacs in Resident Evil: Extinction and Dr. Wesker in the following sequel Afterlife.

*The loyal employee*. In 10 cases, individual scientists acting for a specific employer or principal entirely comply with their superiors’ orders. They appear as pure executors. Often playing only minor roles, the identified cases include Kavita Rao in X-Men: The Last Stand; Henry Wu in Jurassic Park; J.F. Sebastian in Blade Runner; Prof. Werner in Twins; Dr. Mactilburgh in The Fifth Element; Xavier Fitch and Laura Baker in Species; Robert Neville (before the epidemic) and his colleague Alice Krippin in I am Legend, as well as Ilsa Hayden in Judge Dredd.

#### Research teams

In the 35 films, we could identify 11 research teams. Presented as a group, without being given any further personality or character, these researchers mainly execute the orders of either their team leader or their employer. Often not even the names of individual members are mentioned, or only one person is put forward whereas the rest remains in his or her shadow. Scientific teams appear in Spider-Man in the genetics laboratories of Columbia University; in the first episode of Jurassic Park as assistants of Dr. Wu; in Splice as Elsa’s and Clive’s colleagues; as well as in Alien: Resurrection and in The Fifth Element. Except from one or two specific leading person (respectively Dr. Isaacs, Dr. Merrick, Prof. Werner and Dr. Worrington and his assistant Dr. Kavita Rao) not further illustrated research teams are moreover said to be or even partly shown as being involved in the application of bio-engineering technologies in the films Resident Evil Extinction, The Island, Twins or X-Men: The Last Stand.

#### Companies and regimes

Our analysis shows that the main driving force behind the application of SB and other bio-technologies is not always presented as an individual but can also be an institutional body, generally a company, political regime or military institution. Only in Spider-Man this role is taken by a university. In total we have found nine companies in the following films: Code 46 (The Sphinx); the Resident Evil sequels (Umbrella Corporation); Splice (Newstead Pharma); Jurassic Park and The Lost World: Jurassic Park (InGen); The Fly II (Bartok); X-Men: The Last Stand (Worrington); Blade Runner (Tyrell), The Island (Merrick Biotech); and Fantastic Four (Doom Industries).

Governments as major initiator or driver of SB related research appear in four cases: Code 46, Judge Dredd, Avatar and Twins. The army is playing this role in The Fifth Element, Alien: Resurrection; Species; Hulk and The Incredible Hulk.

The main criterion is that institutions are handled like acting and decision taking bodies. Their names are mentioned to designate the main power behind the scientific activities. Thus, the researchers behind them are either not characterised or acting as simple employees. There may be not further specified boards whose members remain anonymous to the audience. In some of the assessed cases, a specific person can be identified as major representative. This may be the company’s owner, such as John Hammond in Jurassic Park, Dr. Worrington in X-Men: The Last Stand, Dr. Eldon Tyrell in Blade Runner and Dr. Merrick in The Island, or the director or board chairperson, such as Dr. Wesker in Resident Evil – Afterlife, Joan Chorot in Splice and Bahkland in Code 46.

### Discussion: The SB scientist in the movies – from old to new clichés

SB is about bringing together science (biology) and engineering techniques. It thereby emphasises a new understanding of combining – more theory based – science and – more practical oriented – technology. This blurring and subsequent fusion into what is called technoscience becomes particularly evident when analysing the metaphors that are mainly used, for instance in the media, not only to present SB as science but also SB biologists (see e.g. Cserer and Seiringer 2009; Hellsten and Nerlich 2011). SB research is often described as playing with Lego bricks or using a construction kit. The world’s most prominent synthetic biologist, Craig Venter, is depicted in public media on his sailing boat (Washington Post 2011) or shown dressed half with a white lab coat and half with a black suit (Business Week Magazine 2004), expressing the image of a businessman-like scientist or entrepreneur.

In some cases, SB may also be presented as “*Do-it-yourself-*”biology. According to this image, the modern synthetic biologist is a “*bio-hacker*” or “*DIY-scientist*” who rejects the idea that science can only be done by academic researchers. He experiments in his own kitchen, garage or amateur lab (Schmidt 2008; Ledford, 2010).

Whether SB engineers are presented as businessmen or as bio-hackers, the image of SB forged by these representations is one that questions the purely academic and scientific nature of the field. Much more, it conveys a picture of SB being driven by an increasingly entrepreneurial and industry-minded spirit.

It has been argued that the popular cultural representations of the scientist has been rather stable throughout the 20th-century, seeing scientists as physically not attractive men with frizzy hair, big glasses and white lab coats who work in secret places. (Frayling 2005). The novel impression of bio-scientists conveyed by the public media however significantly challenges this “stability”. Also appearing in fiction, this new way of portraying SB scientists questions the relevance of traditional ones.

#### The stereotypical scientist

Comparing the portrays of scientists and bioengineers found in the analysed films with – what could be called – “classical” stereotypes placed on scientists in general, it is certainly possible to find a couple of overlaps, although with some exceptions.

A number of surveys conducted in the second half of the 20th-century among the Western society have pointed out a rather uniform and consistent image of scientists (Frayling 2005). According to these studies, scientists in general are commonly seen as “*almost invariable male (…), middle-aged or older, either bald or having a large mass of hair in the style of Einstein*” (Haynes 1994, p.1). This is for instance exemplified by the survey conducted by Roslynn Haynes with regard to common stereotypes in science fiction literature and the drawing reproduced in her book (Haynes 1994, p.1). The notorious and widespread image of the “*mad scientist*” is closely linked to the impression that scientists work “*alone and in isolated laboratories*” and conduct secret or even dangerous research with often critical results. Haynes’ findings mainly confirm results from previous studies, such as notably the pioneering survey Margaret Mead conducted in 1957 among US high school children (Mead and Métraux 1957). The roots of such an image of scientists are old. They can be traced back as far as to the Middle Ages when chemists were portrayed as mad and secretly acting alchemists, wishing to pursue their scientific aims even by crossing moral boundaries (Schummer 2011).

Whereas studies on fictional representation of scientists, such the one carried out by Haynes, in many cases concentrate on literature, the analysis of 222 movies by Peter Weingart shows that the typical cliché image of scientists also prevails in most films (Weingart et al. 2003). According to Weingart’s findings, movie scientists are mainly white/ Caucasian, American, male and middle aged, and little information is provided on their private lives and relationships. If specified at all, they are mainly singles.

Regarding the relevance of these characteristics for the fictional bio-engineers identified in our film analysis, we have to differentiate between those working independently and those who are employed. In the first group, all eight identified independent bioengineers are men. Only among the protagonists in the films added for comparative purposes, two – the geneticist Dr. Claire Wellington in The Stepford Wives and Dr. Hineman in Minority Report – are women.

For the films featuring scientists working as employees, the ratio is a little different. Here, we can find seven female researchers – Elsa in Splice, Dr. Susan McAlester in Deep Blue Sea, Ilsa Hayden in Judge Dredd, Dr. Laura Baker in Species, Dr. Grace Augustine in Avatar, Dr. Alice Krippin in I Am Legend and Dr. Kavita Rao in X-Men: The Last Stand. The number of employed male scientists in these films is also seven: Clive in Splice, J. F. Sebastian in Blade Runner, Dr. David Banner in Hulk, Dr. Xavier Fitch in Species, Dr. Henry Wu in Jurassic Park, Dr. Isaacs in Resident Evil: Extinction and Dr. Wesker in Resident Evil: Afterlife. Also as regards the bio-engineers’ age, independent scientists tend to be older than scientists working as employees. The latter are often presented as mainly overambitious newcomers, whereas single scientists are already more established and experienced.

The portrayed scientists have all white skin colour, except for Dr. Wu and Ilsa who are both of Asian origin, Dr. Neville played by Afro-American Will Smith, and Dr. Rao whose actress is Iranian while the comic book character the role is based on is an Indian geneticist.

Film synthetic biologists and bio-engineers are also mainly unmarried, if this is specified at all, and their relationships or love life are not further elucidated. Exceptions are made by cases where, similar to Frankenstein or Rothwang in Metropolis, scientists have lost their beloved and theses losses significantly impact on their scientific activities, such as in Godsend or in X-Men Origins: Wolverine. Forming a conventional modern couple with an apparently normal private life, Elsa and Clive in Splice present an exception.

Regarding the characteristic “*working alone and in isolated laboratories*”, our analysis has again revealed the importance to distinguish between independently acting and employed SB bio-engineers in films. In the first group, a recurrent stereotype is that of the formerly brilliant scientist who was then excluded from society for his unethical research. This isolation is symbolised by the person’s living and working undercover (David Banner in Hulk), on a remote and secret island (Stryker in X-Men Origins: Wolverine, Dr. Moreau) or outside the big city (Dr. Wells in Godsend). Scientists may hereby appear as somehow weird and mad, megalomaniac and unsocial, emulating thereby the classical image of Dr. Frankenstein. Being most often part of a team, employed SB scientists are, in contrast, generally integrated in their environment. Although, their private lives are usually not much unveiled, they nevertheless appear as normal yet exceptional colleagues. This usually changes if they turn from loyal to rebellious: Elsa and Clive (Splice) are forced to move to the remote farm house when they realise the consequences of their forbidden experiments. However, they try to remain integrated in their research team and to conceal their private research. Also Susan McAllister (Deep Blue Sea) keeps her secret about the genetic manipulated sharks as long as she can, in order not to be expelled by her team.

The sticking in some of the films related to SB to traditional clichés of scientists, especially to that of mad but ingenious scientists, working alone and in isolated places can be explained in different ways.

First, by “*attempting to generate biological systems with new features that might never have existed before as part of any natural living system*” (Boldt and Müller 2008), SB may be perceived by the public as threatening or bewildering (Pühler et al. 2011). New knowledge is generally met with ambivalence (Weingart et al. 2003). Especially the idea of creating artificial life and designing organisms as conveyed by the media in the context of SB is likely to trigger a strong feeling of mistrust and suspicion, notably as it transgresses ethical boundaries and carries the risk of misuse. Taking this into consideration, film makers may therefore opt to coming back to the old picture of the sinister and megalomaniac scientist when depicting scientists applying SB and SB related technologies, forging and strengthening an image that has proved its worth and appears to be commonly accepted .

Second, the public perception and understanding of new technologies are closely linked to old narratives (Nottingham 1999). Several studies emphasise the importance of stories for understanding public perceptions of new technologies, such as for instance regarding nanotechnologies (Davies and Macnaghten 2010; Dupuy 2010; Ferrari and Nordmann 2010). Social and ethical thinking as well as the public debate about science and technology are often based on and enriched by legends and myths. “*Frankenstein Factor*”, “*Frankenstein Science*” or “*Frankenfood*” have become increasingly popular concepts, especially in the media covering emerging bio-technologies like SB and their possible consequences (Miller and Conko 2004; Boldt and Müller 2008). As Schummer moreover highlights, besides allusions to Frankenstein, the production of the first synthetic cell by Craig Venter in 2010 was also set in relation by many newspapers with the Creation myth, Pandora’s Box, Aeolus’ bag or quotes from Goethe’s Faust (Schummer 2011). Referring to already known pictures and stories helps the – non-scientifically educated – audience to domesticate technological progress and findings, and to apprehend and easier morally judge them (Hall 1997 Keppler 2006). If film makers therefore stick to classical stereotypes, with their protagonists reminiscent of characters from the Frankenstein, Golem or Faust narratives, they make use of well-known motives which helps them to easier translate and communicate complex ideas, such as applying engineering to biology.

#### The scientific entrepreneur

The partial attachment to classical stereotypes that we still find in some SB relevant movies is contrasted by a fictional image of scientists who follow a more economic than academic spirit. Films hence reflect a current trend in modern sciences: the accelerated rise of industry-based research (Shapin 2008). In fields such as SB and bio-technologies, this rise is particularly visible. Here, the need for high investments and the lack of public funds, as well as the commercial potential of scientific results have let to the creation of more and more for-profit research companies. As a consequence, the “*scientific entrepreneur*” has emerged as a new type of researcher “*who is both a qualified scientist and, like all commercial entrepreneurs, a risk taker*” (Shapin 2008, p. 210).

Presenting an alternative to the classical Frankenstein cliché, this image is mainly used in the media when portraying synthetic biologists, as has already briefly been underlined above. In October 2008, the popular US Magazine *Esquire* (Esquire 2008) portrayed four synthetic biologists among the 75 most influential people in the 21st-century: besides Craig Venter, Drew Endy (presented in the article as “*Creator of synthetic life*”), Jay Keasling (“*Genetic engineer pioneer*”) and Anthony Atala (“*Builder of human organs*”). The manner *Esquire* presents them in no way reminds of the old fictional stereotype of mad, bad and evil scientists. They are shown as young or middle aged outstanding and promising scientists who, even if affiliated to some university department, have close ties with the industry which funds and uses their work.

Our analysis shows that also film makers tend to turn towards a representation of bio- and genetic engineers that reflects this perception of the reality. However, the figure of the scientific entrepreneur that fully manages his own company is still rather rare in films. We find it in form of John Hammond in Jurassic Park, Dr. Merrick in The Island, Dr. Eldon Tyrell in Blade Runner or Dr. Worrington in X Men. More common in contrast is the character of the scientist who works in a large industrial research laboratory, and, as salaried employee, “*(assumes) little personal risk and commonly (expects) few of the rewards of successful entrepreneurial activity by the company*” he or she works for (Shapin 2008, p. 210). Elsa and Clive (Splice), Dr. Wu (Jurassic Park) and Dr. Rao (X-Men: The Last Stand) but also J.F. Sebastian (Blade Runner) or whole research teams such as in The Island are depicted in this way.

Such a representation of the modern bio-scientist as a typical team member again contrasts the classical stereotypical idea of scientists working alone. In return, it corresponds to real developments as can be seen from several recently conducted studies. Investigating the number of authors per scientific publication, Stefan Wuchty, Benjamin F. Jones and Brian Uzzi for instance demonstrate that scientific knowledge creation is increasingly teamwork (Wuchty et al. 2007). Unlike researchers in the 18th, 19th or early 20th-century, who used to work alone and hereby have contributed to forging the classical stereotype, today’s scientists are increasingly conducting their projects in teams. The authors explain this dominance of collective over individual research in science and engineering primarily by the large scale of most projects, their complexity, as well as high costs (Wuchty et al. 2007) – arguments that also account for research conducted in SB. Christopher Baethge (2008) in this regard compares Charles Darwin, who published his theory of evolution as a single author, with the Human Genome Project which involved more than 160 scientists. If hence in the “real” scientific world, teams are increasingly replacing the “*old ideal of the brilliant researcher working alone*” (Baethge 2008), this trend corresponds to the settings shown in films like Jurassic Park, Deep Blue Sea, Resident Evil: Extinction, Spider Man, Splice, The Island or The Fifth Element.

Finally, the de-personalised way of portraying a big company like the Umbrella company (Resident Evil) as main acting power behind the bio-tech research, with no clear single owner or director, in a way pushes to extremes the idea that profit-oriented industrial research is more and more taking the place of virtuous academic research, as expressed by some contemporary observers (Shapin 2008).

#### Motives and social responsibility

*What motives*? Partly replacing, or at least complementing classical clichés by an image of SB scientists as industry-based researchers rather working in scientific teams than alone and isolated also impacts on what motives are assumed by films behind research and the question of responsibility.

In a classical setting, we may find film scientists acting on their own and thereby reminding of Dr. Frankenstein, as regards the motives behind their work: Initially pursuing good intentions, e.g. stopping diseases and pain or offering immortality to humanity, they show traits of ambivalence. If they do not either lose control over their findings or get manipulated or corrupted by third parties, they tend to turn by themselves to subversive, obsessed or megalomaniac persons (Haynes 1994; Weingart et al. 2003). Their motives hence change from benevolence to altruism. Fictional scientists portrayed as employees find themselves in a different position. Even if initially driven by idealism too, these scientists are shown as being bound by the – economic – interests of their employers. Here again parallels can be made to reality perceptions. The survey conducted by Shapin (2008) with some 40 scientists working either in an academic or industrial context unveils some stereotypical perceptions. Compared to their colleagues at university, industry-based researchers are often considered as having less freedom, with clear commercial and profit-oriented motives standing behind their work. A similar impression is conveyed by several of the analysed films. Some films’ stories even develop out of the conflict of interest that arises for industry-based scientists who are unable to combine their scientific curiosity with their employer’s purely profit-oriented goals. This ambivalence of scientists caught between their own scientific interests and market-oriented research agendas may, however, be related to another classical myth: the one of the sorcerer’s apprentice who ignoring his master’s orders and following his own curiosity causes a catastrophe – by animating his broom.

*Who takes the responsibility?* The choice of either presenting SB scientists as acting on their own or as industry employees also reflects on how the issue of responsibility is handled in a film. Whether researchers can be held responsible for scientific findings or, in contrast, technologists and politicians who apply them is a much debated question, especially since the Second World War and the Nuclear Age (Koepsell 2010). Many science fiction films address the “*questions about moral responsibility for research*” and “*decisions about how and by whom knowledge should be owned, controlled, and implemented*” (Haynes 2003, p. 252). Roslynn Haynes (2003) notes in this regard that the way this question about the scientists’ responsibility for technological risks has regularly been addressed by the society has also been reflected in several science fiction films featuring megalomaniac scientists. The classical film scientist is hence described as being driven by “*paranoia, delusions of grandeur, obsessive behaviour*”, which finally makes him appear as the only responsible for what he has invented and developed (Haynes 2003, p. 252). This impression also applies to those fictional SB engineers who still personify the old clichés of the megalomaniac scientist. In the analysed films, individually acting scientists, like Dr. Moreau, Stryker or Dr. Wells are mainly presented as being responsible for the impact of their action, including the newly designed products’ (mis-)behaviour. Similar to what Schummer (2008) notes regarding classical clichés, these fictional scientists do not show any form of regret. Their death at the end of the film may hence symbolize some sort of justice.

Regarding the modern approach to depicting bioengineers in films, the situation is quite different, depending on whether the action can be related to a single scientist, a team or an institutional body. Being mainly shown as integrated into their social environment, most individually portrayed SB scientists sooner or later become aware of their going too far and finally at least try to act in a responsible manner. Initially driven by their untamed scientific curiosity and zeal, Elsa in Splice and Susan in Deep Blue Sea are starting their projects in the belief that their pioneering findings will bring benefit to mankind. They both however know that they exceed moral and ethical limits. In this sense, they remind of Dr. Frankenstein in the 1994 adaptation: although portrayed as being megalomaniac and immoral, Frankenstein also aims at finding cures to major diseases or even paving the way towards immortality. In view of the provoked catastrophe, Elsa and Susan however assume responsibility and take all efforts to destroy their inventions. The image conveyed by these examples is hence that of a scientist who, although still showing some traits of megalomania or excessive zeal, is convinced to cross boundaries for the good of humanity, and finally accepts to take charge of the damage caused. They hereby reflect what Dinello calls “a more positive strain of science fiction scientists, (…) who try to help humanity, but who accidentally create a technological threat to themselves, their family, the society, or even the universe. These misguided scientists, oblivious to the consequences of their work, possess positive motives and try to rectify their mistakes” (Dinello 2005). If thereby contradicting the intentional immoral behaviour of many classical fictional scientists, as discussed by Haynes or Schummer, these scientists nevertheless show traits of Dr. Frankenstein who at the end travels around the world to kill his monster, or Dr. Delambre in the original version of The Fly from 1958, who unlike Brundle in the film’s 1986 remake, finally asks his wife to kill him and destroy his invention.

In contrast, if synthetic biologists form a team, the question of who takes the responsibility is most often not sufficiently addressed and remains open. In Jurassic Park, for instance, the main researchers even leave the island for the weekend prior to the catastrophe. They thereby indirectly demonstrate their denial of any responsibility for what they have produced in their test tubes.

A similar scenario may apply when the main agent behind SB research is a company, the government or the army, with no clear front person. These institutions may either delegate the responsibility to one of their employees, such as in Resident Evil: Extinction where Dr. Isaacs is assigned to find a remedy for the virus and stop the spreading Zombie epidemic. Or they completely ignore the responsibility they have, both for their inventions and the damages these may do. Again, in Jurassic Park, a concern over responsibility is raised only once, when one of the invited researchers, Ian, states that the park’s director and owner, Hammond, cannot take responsibility for what his company InGen has achieved because he has only built on research outcomes that already existed.

The way of dealing with responsibility, by either not clearly identifying or holding responsible the main culpable of the catastrophe generated by new bio-technological findings and their application is nurturing the general debate about ethical concerns in the context of bio-technologies, the boundaries they may cross and the need for regulations. Showing a fictive situation where SB engineers act in a manner that from today’s point of view is controversial and prove either unable or unwilling to assume responsibility for any negative consequence, films contribute to forging either an image of scientists evading their responsibility in ethically controversial questions or an impression of a complex scientific environment that makes it impossible to still identify the main responsible. By proposing concrete, although fictive examples and stories, films certainly intervene in the public debate and may, consequently, influence their audience’s position in ethical and social issues raised in the context of SB research. Public media, for instance, reporting on this debate, often opt to do so by referring to Hollywood examples. Just to give an example: Discussing in June 2010 the breakthrough made by the Craig Venter Institute and its possible consequences, the German newspaper Die Zeit started its article with illustrations from the film Splice and the question “What happens if the bio-industry succeeds in re-programming the human body?”.

#### The absence of the bio-hacker

The recent development of SB has triggered the emergence of a growing community of amateur biologists wishing to apply SB technologies, assemble DNA or rebuild whole viruses in their mobile labs, garage or kitchen (Wohlsen 2011 Schmidt et al. 2009b). These often called bio-hackers or bio-punks are mainly “*college student(s) eager to demonstrate their technological prowess*” (Kelle 2009, p.104). In the analysed movies, such an “*amateur*” scientist with his or her “*homemade*” synthetic organism cannot be found. The image of the SB scientist as “*Do-it-yourself biologist*” seems to be completely absent from the high gross box-office movies. Even if labs are located neither in an academic nor an industrial but rather a private environment, such as those of Stryker, Seth Bundle and Dr. Moreau, they do not look like what could be called a low-budget garage or amateur workshop. Their workplaces are highly sophisticated and high-tech labs. The movies therefore do not convey the image of SB as new playground for bio-hackers. The only DIY biologist example may be found in one of the comparative films: In the 1994 adaptation, young student Frankenstein is constructing his animated monster in his loft-like student digs where he has gathered different equipment for his experiment.

## Conclusion

Whereas the myth of Frankenstein is ubiquitous when public journals and newspapers cover the recent developments in SB, the way scientists applying SB or SB related technologies are characterised in movies is questioning the relevance of classical and preconceived stereotypes. Film makers seem to be influenced by real trends and tendencies that affect the way bio-sciences are done nowadays. The old male genius but mad scientist who serves as model in many science related movies cannot necessarily be found in films that take up concepts and ideas close to SB. As a clear alternative, film SB bio- and genetic engineers tend to be depicted as modern researchers with close ties to the industry or employed by large bio-tech firms. Their research is hence driven by a more entrepreneurial and market-driven than academic spirit. Still inspired by a similar zeal to play God, overcome death and create life than his classical prototype, Frankenstein 2.0. differs by being better integrated into his social environment. Involved in market-oriented research, he or she is more reflecting the image of a scientific entrepreneur than that of a weird megalomaniac professor. Taking this idea one step further, film makers also tend to see a powerful company, political regime or army as main driver of SB research.

By forging the image of science as entrepreneurial business with company owners and salaried scientists, films in a way discuss and question the benefits of neoliberal business practices in the scientific world, meaning practices that follow a profit, commercial and market oriented rather than a purely scientific approach. If research is shown as being primarily driven by economic interests, the goal becomes the production and selling of scientific knowledge and findings for profit maximization, sometimes even by crossing ethical boundaries. As a consequence, employed scientists either appear as “workers” fulfilling their company’s business goals, or as being torn between the expected loyalty and profit-oriented commitment and their own research ambitions.

Closely linked hereto, the current way of showing scientists engaged in SB and related research has also to be seen within the actual debate about emerging bio-technologies. By taking up aspects and stereotypes already increasingly emphasised in the media and showing scientists as being either primarily profit-driven or a small wheel in a large machinery, films also provide potential illustrative inputs to the debate on ethical and social concerns related to bio-sciences, their potential to cross moral boundaries and the question about who is finally taking responsibility. Showing scientists in a rather realistic way and quite similar to how they are depicted in the media, scenarios are likely to offer at least partly “imaginable” stories and possible illustrations for the public debate. If the white-bearded professor we have in fact never seen in real life is replaced by the young, intelligent and dynamic, yet seemingly “normal” scientist, the pretext or own reassurance of “this is only a story” may appear less evident.

In conclusion, we can say that Frankenstein 2.0. seems to refute the old cliché of the bad and mad scientist. In return, he is contributing to forging a new one: that of a modern and contemporary synthetic biologist whose academic excellence is challenged either by his own or his superiors’ entrepreneurial spirit.

Understanding this image and the message it conveys is indeed crucial to anticipate how SB and related emerging technologies that still remain little known to the general public will finally be perceived and generally accepted.

## Endnote

^a^The project CISYNBIO is implemented by IDC under the Austrian Research Programme Gen-Au (Austrian Federal Ministry for Science and Research) over the period August 2009 to August 2012. More information can be found on the project’s website: http://www.cisynbio.com and IDC’s website http://www.idialog.eu.

## Electronic supplementary material

Additional file 1: Table S1: List of selected and analysed films. (DOC 66 KB)
