# Supplementary material for: Frankenstein 2.0.: Identifying and characterising synthetic biology engineers in science fiction films
Source: Life Sci Soc Policy. 2013 Oct 1;9:9. doi: 10.1186/2195-7819-9-9 (PMC4513001; doi:10.1186/2195-7819-9-9)
Supplement: Supplementary file 1 — Additional file 1: Table S1: List of selected and analysed films. (DOC 66 KB) [file 40504_2013_9_MOESM1_ESM.doc]

Additional file 1: List of selected and analysed films

**Table S1**: List of selected and analysed films, including production year, project internal classification and box office value. (SB: Synthetic biology related film, COMP: comparative film)

| **Film title** | **Production Year** | **SB related or used for comparison** | **box office value (**in US$**)*** |
| --- | --- | --- | --- |
| Golem, wie er in die Welt | 1920 | COMP | n.n. |
| Metropolis | 1927 | COMP | 650.422 |
| Island of Dr. Moreau, The ^x^ | 1977 | SB | 6.000.000 |
| Blade Runner | 1982 | SB | 33.139.618 |
| Star Trek3: Search for Spock | 1984 | SB | 87.000.000 |
| Fly, The | 1986 | SB | 40.500.000 |
| Twins | 1988 | SB | 216.600.000 |
| Fly 2, The | 1989 | SB | 20.000.000 |
| Jurassic Park | 1993 | SB | 923.067.947 |
| Frankenstein | 1994 | COMP | 112.006.296 |
| Gattaca | 1994 | COMP | 15.900.000 |
| Jugde Dredd | 1995 | SB | 113.487.912 |
| Species 1 | 1995 | SB | 113.354.449 |
| Island of Dr. Moreau, The | 1996 | SB | 42.000.000 |
| Alien: Resurrection | 1997 | SB | 160.700.000 |
| Fifth Element, The | 1997 | SB | 263.900.000 |
| Jurassic Park: Lost World: | 1997 | SB | 786.686.679 |
| X Files: Fight the Future | 1998 | SB | 189.176.423 |
| Species 2 | 1998 | SB | 26.817.565 |
| Godzilla | 1998 | COMP | 379.014.294 |
| Deep Blue Sea | 1999 | SB | 165.048.228 |
| Bicentennial Man | 1999 | COMP | 87.420.776 |
| Hollow Man | 2000 | COMP | 191.200.000 |
| X-Men | 2000 | COMP | 334.627.820 |
| Jurassic Park 3 | 2001 | SB | 365.900.000 |
| Blade II | 2002 | SB | 150.145.152 |
| Spider-Man | 2002 | SB | 821.708.551 |
| Minority Report | 2002 | COMP | 358.824.714 |
| Code 46 | 2003 | SB | 197.148 |
| Hulk | 2003 | SB | 245.360.480 |
| Godsend | 2004 | SB | 16.910.708 |
| Spider-Man 2 | 2004 | SB | 786.686.679 |
| I Robot | 2004 | COMP | 348.601.023 |
| Stepford Wives, The | 2004 | COMP | 96.221.971 |
| Fantastic Four | 2005 | SB | 330.579.719 |
| Island, The | 2005 | SB | 163.018.913 |
| X-men: The last stand | 2006 | SB | 459.359.555 |
| I am Legend | 2007 | SB | 585.055.701 |
| Resident Evil: Extinction | 2007 | SB | 148.403.704 |
| Spider-Man 3 | 2007 | SB | 890.871.626 |
| Babylon A.D. | 2008 | SB | 70.216.497 |
| Incredible Hulk, The | 2008 | SB | 263.900.000 |
| Avatar | 2009 | SB | 2.723.292.481 |
| Splice | 2009 | SB | 22.135.508 |
| X-Men Origins: Wolverine | 2009 | SB | 374.825.760 |
| Repo Men | 2009 | COMP | 18.409.891 |
| Surrogates | 2009 | COMP | 119.668.350 |
| Resident Evil: Afterlife | 2010 | SB | 296.221.566 |

* Unless otherwise specified, source: [http://www.worldwideboxoffice.com](http://www.worldwideboxoffice.com/). Not adjusted for inflation. Data collected in spring 2011.

^x^ Estimate. Source: [www.imdb.com](http://www.imdb.com/)
